# From diet to brain repair: natural bioactive compounds in post-ischemic stroke recovery

**DOI:** 10.3389/fnut.2026.1778396

**Published:** 2026-03-27

**Authors:** Fan Bu, Zicheng Zhang, Shaohua Qi, Longsheng Xu

**Affiliations:** 1 Department of Neurology and Psychology, The Fourth Clinical Medical College of Guangzhou University of Chinese Medicine, Shenzhen Traditional Chinese Medicine Hospital, Shenzhen, Guangdong, China; 2 Department of Radiotherapy, Shenzhen Nanshan People's Hospital, Shenzhen, Guangdong, China; 3 Department of Neurology, McGovern Medical School, The University of Texas Health Science Center at Houston, Houston, TX, United States; 4 Department of Anesthesiology and Pain Medicine, The Affiliated Hospital of Jiaxing University, Jiaxing, Zhejiang, China

**Keywords:** food-derived compounds, ischemic stroke, molecular mechanisms, natural bioactive compounds, therapeutic interventions

## Abstract

Stroke represents the leading cause of disability and mortality worldwide, often resulting in long-term neurological deficits, extensive neuronal damage and inflammatory cascades. Ischemic stroke, which accounts for about 80% of stroke cases, is characterized by the sudden loss of blood circulation to an area of the brain, resulting in a corresponding loss of neurologic function. The blood supply interruption induced oxidative stress, mitochondrial dysfunction, neuroinflammation, and gut dysbiosis are involved in complex interactions within brain tissues. Moreover, the reperfusion induced inflammation produces more severe damage compared to the blood supply interruption. Current therapeutic interventions face critical limitations including narrow treatment windows, restricted patient eligibility, and significant adverse effects, underscoring the urgent need for safe, effective adjunctive strategies applicable during extended recovery periods. Recent research highlights the potential of nature biologically active substances, here we referred to food-derived and natural bioactive compounds, as promising therapeutic agents for post-stroke recovery. Administration of these compound by dietary route has the potential to support cellular repair processes via reducing oxidative stress, modulating neuroinflammation, promoting neurogenesis, inhibiting ferroptosis, and enhancing synaptic plasticity. This review examines the current evidence and emerging concepts on the roles of these bioactive compounds in post-stroke recovery and synthesizing mechanistic evidence. We discussed specific dietary sources and pharmacokinetics of selected compounds, providing insights into their bioavailability and potential synergistic effects with conventional therapies. Additionally, we examined clinical studies and evaluated the efficacy and safety of these interventions, offering a translational perspective on their integration into post-stroke rehabilitation. These findings underscore the therapeutic potential of dietary bioactive compounds as adjunctive treatments in post-stroke recovery and highlight the need for dose–response optimization, biomarker-guided precision nutrition approaches for patient stratification, and large-scale trials to validate long-term efficacy and safety in diverse stroke populations.

## 1 Introduction

Ischemic stroke is the second leading cause of death and long-term disability worldwide, with global incidence numbers increasing ~4.07 million in 1990 to ~7.8 million in 2020 (1). Stroke predominantly affects individuals over 65 years of age, though its incidence in younger adults (18–50 years) has been increasing, accounting for approximately 10–15% of all stroke cases (2, 3). It is caused by thrombosis or embolism within the blood vessel and interruption of blood flow, which results in suddenly experiencing paralysis, impaired speech, or loss of vision. The pathophysiology of ischemic stroke involves a cascade of events, including energy failure, excitotoxicity, oxidative stress, inflammation, and cell death, which collectively contribute to tissue damage and functional impairment (4). The brain’s response to ischemic injury includes multiple adaptive mechanisms aimed at limiting damage and promoting repair. Among these, neuroplasticity plays a crucial role in functional recovery by enabling the reorganization of neural circuits and the formation of new connections. However, the endogenous capacity for repair and regeneration is often insufficient to fully restore lost functions, highlighting the need for therapeutic interventions that can augment these processes. The post-stroke recovery strategies can alleviate many symptoms caused by neuron death and inflammation; and there are few interventions to restore the functional neuron circuitries (5, 6). Importantly, once a stroke has occurred, the risk of recurrence and subsequent cognitive impairment increases significantly (7–9). In addition, clinical manifestations and recovery trajectories vary considerably among patients, complicating the development and implementation of uniformly effective therapeutic strategies. Consequently, increasing research efforts are directed toward elucidating the underlying molecular mechanisms that influence long-term outcomes.

Although stroke prevention remains highly effective, it is often more complex to implement at the population level than interventions initiated after disease onset. With advances in acute stroke management, there are limited treatment options available, many patients experience incomplete recovery and persistent neurological deficits. The current therapeutic strategies include intravenous thrombolysis and mechanical thrombectomy can improve outcomes, however, this treatment is highly time-dependent and often limited by delays in recognition, transport, and treatment (10). While acute interventions like particularly intravenous thrombolysis can improve outcomes if administered quickly after the symptom onset, however, earlier administration of thrombolytic therapy usually failed due to the insufficient of rapid recognition of ischemic stroke, and the time-sensitive thrombolysis is not always accessible (11, 12). Recent research has shed light on the potential of various endogenous metabolites in influencing post-stroke recovery. For instance, Petersson et al. demonstrated that ischemic stroke causes significant metabolomic profile alterations in the urine samples, there is a dramatic dysregulation of phenylalanine metabolism, tyrosine metabolism, purine metabolism, glycerophospholipid metabolism, phenylalanine, tyrosine, and tryptophan biosynthesis (13). Their findings suggest that alterations in acylcarnitine levels may have important implications for urine-based metabolomic signatures as biomarkers for stroke, as well as for the development of targeted therapeutic interventions. Meanwhile, the latest review paper also emphasized the correlations between neuroinflammation and energy metabolism, focusing on the inflammatory cytokines and the activation of immune cells, extend to energy metabolism dysregulation and neural cell damage (14). Research on stroke pathophysiology has demonstrated that lifestyle modifications, including a healthy diet, regular physical activity, smoking cessation, and moderated alcohol intake, play crucial roles to reduce the risk of stroke (15, 16). While enhancing neurological recovery, cognitive function, and quality of life after stroke is increasingly recognized as equally-if not more important-in many clinical and personal contexts than prevention alone. Stroke survivors often face lasting impairments in motor control, cognition, speech, and emotional regulation, all of which profoundly affect independence and life satisfaction; therefore, the effective intervention of post stroke impairment is essential to ensuring treatment efficacy (17, 18). Therefore, we have chosen to draw attention especially to find rational, innovative neuroprotective compounds to stroke recovery not only reduces mortality and disability but also alleviates the long-term economic and social impact of stroke on individuals and healthcare systems.

Food-derived and natural bioactive compounds-particularly those that can be realistically incorporated into dietary patterns-have emerged as promising adjunctive strategies for promoting stroke recovery, owing to their capacity to modulate key biological processes involved in neurorepair. These naturally occurring substances-such as polyphenols (e.g., curcumin, resveratrol), omega-3 fatty acids (e.g., DHA, EPA), and sulforaphane from cruciferous vegetables exert antioxidant, anti-inflammatory, anti-apoptotic, suppression of ferroptosis, and neuroprotective effects that help limit secondary brain damage and support neural regeneration. The effect of these components is independent of their nutrition and energetic importance, and their existence also limited to specific plant foods, such as fruits, vegetables, cereals, grains. For example, curcumin [enriched in turmeric (*Curcuma longa*)] has been shown to suppress post-stroke inflammation by downregulating NF-κB signaling, while resveratrol enhances angiogenesis and neurogenesis through activation of the Sonic Hedgehog signaling (19). Omega-3 fatty acids help maintain membrane integrity and promote synaptic plasticity, crucial for motor and cognitive recovery (20). Additionally, compounds like ginsenosides and berberine may stabilize the blood–brain barrier and modulate microglial activation (21, 22). These bioactive components work through multi-target mechanisms, making them attractive as safe, dietary-based strategies to complement pharmacological treatments and rehabilitation in post-stroke care. Ongoing research and clinical trials continue to explore their therapeutic potential in improving long-term outcomes for stroke survivors.

Here, we focus on the potential of bioactive compounds in promoting neuroplasticity, reducing inflammation, ferroptosis, and enhancing functional recovery after ischemic stroke. The scope includes bioactive compounds achievable through realistic dietary intake and purified phytochemicals or concentrated plant extracts-including those derived from traditional Chinese medicine (TCM). We synthesize current evidence on the role of food-derived and natural bioactive compounds in post-ischemic stroke recovery. Literature searches were conducted in PubMed, Web of Science, and Scopus databases, focusing on publications from 2015 to 2025, with seminal earlier works included where appropriate. We prioritized studies providing mechanistic insights into compound actions, with emphasis on preclinical models demonstrating clear pathophysiological relevance and clinical studies reporting functional outcomes. Both *in vitro* and *in vivo* experimental evidence were evaluated, along with available clinical trial data. Given the rapidly evolving nature of this field, particular attention was paid to recent advances in understanding molecular mechanisms, including ferroptosis, gut-brain axis interactions, and nutrigenomic effects.

In this review, we summarized the molecular mechanisms underlying ischemic stroke pathophysiology and critically evaluated limitations of current pharmacological interventions. Additionally, we systematically analyzed the natural bioactive compounds demonstrating neuroprotective efficacy in preclinical stroke models. Furthermore, we also explored the challenges and opportunities in translating preclinical findings to clinical applications, including issues related to bioavailability, dosing, and potential interactions with conventional stroke therapies. By providing a comprehensive analysis of current literature and identifying key knowledge gaps, this review aims to inform future research directions and accelerate the development of evidence-based, precision nutrition approaches for post-stroke recovery.

## 2 Pathogenesis of ischemic stroke and post-stroke recovery

The etiology of ischemic stroke is clarified as non-modifiable and modifiable, non-modifiable risk factors include the genetic, aging, ethnic, and gender, while the modifiable risk factors consist environmental trigger and lifestyles (23, 24). These factors interact and cause cerebral blood vessel alteration and vulnerability, once the intravascular pressure breaks through the threshold or blocks, the blood supply suddenly interrupts. Ischemic brain injury triggers a cascade of interrelated pathological mechanisms that contribute to neuronal death and long-term neurological deficits, and finally results in permanent brain damage, disability, or death. One of the earliest and most damaging events is excitotoxicity, where oxygen and glucose deprivation lead to excessive release of glutamate and over-activation of NMDA and AMPA receptors, causing calcium influx and neuronal hyperexcitation (25). This sets off oxidative stress, as mitochondrial dysfunction and enzyme activation generate large amounts of reactive oxygen and nitrogen species (ROS/RNS), damaging proteins, lipids, and DNA. In parallel, inflammatory responses are rapidly initiated, with activation of microglia, infiltration of peripheral immune cells, and production of pro-inflammatory cytokines like TNF-α and IL-1β, further exacerbating tissue damage (26). These combined insults drive apoptotic and necrotic pathways, including caspase activation and mitochondrial cytochrome C release, leading to programmed cell death (27). Additionally, disruption of the blood–brain barrier (BBB) occurs through endothelial injury, tight junction breakdown, and matrix metalloproteinase activation, which increases vascular permeability and contributes to cerebral edema and secondary injury (28). Together, these mechanisms form a self-amplifying loop of neurotoxicity, highlighting the need for multi-targeted therapies to effectively protect and repair the ischemic brain. Moreover, these mechanisms are tightly interconnected and evolve over time, making them critical targets for therapeutic intervention in acute stroke management.

Stroke progression is generally divided into three phases-acute, subacute, and chronic-each defined by distinct pathophysiological mechanisms and clinical priorities (29). The acute phase, occurring within the first hours to days after onset, is characterized by rapid neuronal injury resulting from abrupt interruption of cerebral blood flow. Deprivation of oxygen and glucose triggers a cascade of biochemical events, including excitotoxicity, mitochondrial failure, oxidative stress, and inflammation. A rapid increase in ROS further amplifies cellular damage (30). The primary therapeutic objective during this phase is timely reperfusion-achieved through thrombolysis or mechanical thrombectomy-to restore cerebral blood flow and limit infarct expansion. However, reperfusion itself may exacerbate injury by generating an additional surge of ROS, a phenomenon known as reperfusion injury, which can worsen oxidative damage and compromise early recovery (31, 32).

The subacute phase, spanning days to weeks after the initial insult, represents a critical transitional window between acute injury and long-term remodeling. During this period, the ischemic penumbra-the hypoperfused yet structurally viable tissue surrounding the infarct core-remains metabolically unstable but potentially salvageable. Unlike the necrotic core, which undergoes irreversible energy failure, penumbral neurons experience sustained metabolic stress, delayed apoptosis, mitochondrial dysfunction, oxidative accumulation, and emerging ferroptosis signaling. Microglia play a central regulatory role in this phase. Initially polarized toward a pro-inflammatory phenotype characterized by TNF-α, IL-1β, IL-6, and ROS production, microglia can exacerbate secondary injury and contribute to blood–brain barrier disruption. As the subacute phase progresses, however, a subset transitions toward a reparative phenotype expressing Arg1, CD206, IL-10, and neurotrophic factors such as BDNF and GDNF. This phenotypic plasticity is particularly evident in the penumbra, where the balance between pro-inflammatory and pro-regenerative signaling influences whether tissue undergoes delayed degeneration or functional recovery. Consequently, the subacute stage represents a pivotal therapeutic window for interventions aimed at limiting secondary degeneration, modulating oxidative and iron-dependent (ferroptotic) injury cascades, and promoting neuroplasticity.

The chronic phase, which may extend from months to years, is characterized by stabilization of the lesion and long-term remodeling of neural networks. Functional outcomes during this stage depend on the extent of initial injury, the integrity of residual circuitry, and the effectiveness of rehabilitation and restorative interventions.

## 3 Therapeutic strategies for stroke

### 3.1 Attenuation of oxidative stress pathways

The brain is particularly vulnerable to oxidative stress due to its high oxygen consumption, abundant lipid content, and comparatively limited antioxidant defenses (33). Under physiological conditions, redox homeostasis is maintained by coordinated endogenous antioxidant systems, including the Nrf2-Keap1 signaling pathway, the glutathione (GSH) system, superoxide dismutases (SOD1 in the cytosol and SOD2 in mitochondria), catalase, glutathione peroxidases (GPXs), and the thioredoxin/peroxiredoxin network (34). Nrf2 functions as a master regulator of antioxidant defense, inducing transcription of genes involved in GSH synthesis (GCLC, GCLM), NADPH regeneration, and detoxification of ROS (35, 36). Mitochondria possess intrinsic antioxidant defenses that limit superoxide accumulation and preserve membrane integrity. Together, these systems maintain physiological redox balance and protect neuronal function.

During ischemic stroke, however, these protective mechanisms become overwhelmed or impaired. Interruption of cerebral blood flow results in oxygen and glucose deprivation, leading to ATP depletion and mitochondrial dysfunction, ultimately resulting in neuronal death and dysfunction (37, 38). Impaired electron transport chain activity drives excessive ROS production, while activation of NADPH oxidases further amplifies oxidative burden. Reperfusion exacerbates this injury by generating additional bursts of ROS and RNS, a process known as reperfusion injury (39–41). Simultaneously, depletion of intracellular GSH, dysregulation of Nrf2 signaling, and iron-dependent lipid peroxidation weaken endogenous defenses, promoting neuronal death and contributing to ferroptosis pathways. Oxidative damage extends beyond the acute phase, disrupting the BBB, impairing vascular integrity, and activating inflammatory cascades that perpetuate secondary injury.

Clinical studies indicate that elevated oxidative stress markers persist into the subacute and chronic phases of stroke, potentially limiting neuroplasticity and functional recovery (42). Sustained redox imbalance has been associated with poorer neurological outcomes and increased risk of post-stroke complications, including cognitive impairment and depression. Given the central role of oxidative stress in both acute injury and long-term remodeling, modulation of redox pathways represents a critical therapeutic objective (43).

Although conventional antioxidant supplementation has yielded inconsistent results in clinical trials, increasing attention has focused on bioactive plant-derived compounds capable of modulating endogenous antioxidant pathways rather than merely scavenging free radicals. Preclinical and emerging clinical evidence suggests that such compounds may reduce ischemia-induced damage through multifaceted mechanisms, including activation of Nrf2 signaling, preservation of mitochondrial function, suppression of inflammatory responses, inhibition of apoptosis, improvement of microcirculation, and epigenetic regulation. Importantly, their neuroprotective effects extend beyond simple ROS neutralization (44–46).

Complementary therapeutic approaches such as hyperbaric oxygen therapy (HBOT) have also demonstrated beneficial clinical effects, particularly during acute and rehabilitation phases, although underlying mechanisms remain incompletely defined (47, 48). Transcriptomic analyses in middle cerebral artery occlusion (MCAO) models indicate that HBOT modulates inflammatory signaling, blood–brain barrier integrity, neural repair pathways, and RNA epigenetic modifications, including reduced m6A methylation levels (49). These findings suggest that redox modulation may interact with epigenetic regulation to influence post-ischemic neuroprotection and recovery.

Collectively, disruption of endogenous antioxidant systems and persistent oxidative stress represent central features of stroke pathology. Strategies aimed at restoring physiological redox regulation-through both dietary bioactive compounds and adjunctive therapies-may help preserve neuronal integrity and enhance functional recovery.

### 3.2 Anti-inflammatory effects

Inflammation is another critical factor in stroke pathophysiology, contributing to both acute injury and long-term neurodegeneration. The inflammatory response is rapidly activated after ischemic injury. Microglia, the brain’s resident immune cells, become activated and release pro-inflammatory cytokines such as TNF-α, IL-1β, and IL-6 (50, 51). Peripheral immune cells, including neutrophils and monocytes, infiltrate the brain through a compromised BBB, exacerbating tissue damage (52). While inflammation is part of the repair process, excessive or prolonged inflammation can worsen outcomes. Regulatory T cells, for instance, have been identified as key players in promoting recovery after ischemic stroke by suppressing excessive inflammation and creating a pro-regenerative tissue environment (53). Understanding these temporal phases is essential for tailoring therapeutic strategies to optimize both acute neuroprotection and long-term neurorehabilitation.

The role of inflammation in stroke pathophysiology and recovery has been well-established, and both endogenous metabolites and exogenous supplementary have shown promise in modulating inflammatory responses. Certain bioactive compounds found in foods have demonstrated the ability to influence immune cell function and inflammatory signaling, suggesting potential synergies with endogenous immunomodulatory mechanisms. Many plant-derived bioactive components possess anti-inflammatory properties that could potentially modulate the inflammatory response following stroke. Flavonoids, for example, have been shown to inhibit pro-inflammatory cytokine production and reduce microglial activation, which are key processes in stroke-induced neuroinflammation (54, 55). A cross-sectional study analyzed the data from data from the National Health and Nutrition Examination Survey for 3,675 participants (aged 60 and older) to investigate relationships between dietary flavonoids and stroke risk, the results suggesting that flavonoid-rich dietary patterns are protective against stroke in older adults (56).

### 3.3 Inhibition of apoptotic pathways

Apoptosis, a tightly regulated form of programmed cell death, plays a pivotal role in the ischemic penumbra-the metabolically compromised yet potentially salvageable region surrounding the infarct core (57, 58). Unlike necrosis, which is immediate, uncontrolled, and irreversible, apoptosis is a delayed process that can be triggered by multiple stressors, including calcium overload, oxidative stress, and excitotoxic signaling. Once initiated, apoptosis involves a cascade of events such as mitochondrial cytochrome c release, activation of caspases, and fragmentation of nuclear DNA (59). Central regulators of this pathway include the Bcl-2 family of proteins, which orchestrate mitochondrial membrane integrity, and the caspase family of proteases, which execute the death program. Importantly, because apoptotic death unfolds over hours to days, penumbral neurons remain vulnerable yet amenable to rescue during this window.

Therapeutically, targeting anti-apoptotic pathways has shown promise in both experimental and early translational studies. Caspase inhibitors such as z-VAD-fmk and minocycline have been demonstrated to reduce infarct size and improve outcomes in preclinical stroke models (60, 61). Agents that upregulate Bcl-2 or mimic its protective function, such as Bcl-2 overexpression vectors and BH3 mimetics, help preserve mitochondrial integrity (62). In addition, mitochondrial stabilizers like cyclosporine A, which inhibits mitochondrial permeability transition pore opening, and antioxidant compounds such as MitoQ have been tested for their ability to attenuate oxidative stress and apoptosis (63, 64). Although most of these strategies remain experimental, they highlight the therapeutic potential of modulating programmed cell death to salvage penumbral tissue and improve recovery following ischemic injury.

### 3.4 Neurogenesis and synaptic plasticity

Following a stroke, the brain initiates a complex process of neurogenesis and synaptic plasticity as part of its intrinsic attempt to repair damaged neural circuits and restore lost functions (65, 66). Neurogenesis, primarily occurring in the subventricular zone and the subgranular zone of the hippocampus, involves proliferation, migration, and differentiation of neural stem cells into mature neurons. After ischemic injury, stroke-induced signals such as hypoxia, inflammation, and growth factors (e.g., BDNF, VEGF) stimulate this regenerative response. Concurrently, synaptic plasticity-the brain’s ability to strengthen or rewire synaptic connections, is upregulated, facilitating functional recovery by reestablishing communication between surviving neurons (67, 68). Mechanisms such as dendritic sprouting, synaptogenesis, and long-term potentiation are enhanced in peri-infarct regions, supported by astrocyte-neuron interactions and activity-dependent remodeling. However, this endogenous repair is often insufficient or short-lived, especially in aging or severely injured brains. Therefore, therapeutic strategies that amplify neurogenesis and synaptic plasticity-such as physical rehabilitation, pharmacological agents, neuromodulation, and stem cell therapies-are actively being explored to maximize recovery and improve outcomes after stroke (69).

### 3.5 BBB protection

The integrity of the BBB is compromised during ischemia due to endothelial damage, tight junction breakdown, and inflammatory mediator release (70, 71). This disruption allows plasma proteins, immune cells, and potentially neurotoxic substances to enter the brain parenchyma. BBB breakdown not only exacerbates edema and inflammation but also increases the risk of hemorrhagic transformation, especially in patients receiving thrombolytic therapy (72).

Under normal conditions, the BBB tightly regulates the exchange of molecules and cells between the bloodstream and the brain, maintaining homeostasis and protecting neural tissue. However, following a stroke, the rapid onset of oxygen and glucose deprivation leads to endothelial cell dysfunction, degradation of tight junction proteins (such as claudins and occludins), and activation of matrix metalloproteinases (MMPs), particularly MMP-9 (73, 74). These events increase BBB permeability, allowing infiltration of immune cells, plasma proteins, and neurotoxic substances into the brain parenchyma. The resulting vasogenic edema, inflammation, and oxidative stress further exacerbate neuronal damage and hinder recovery. BBB disruption also creates a hostile environment that impairs neurovascular repair and contributes to long-term cognitive and functional decline (75). Understanding the molecular pathways underlying BBB breakdown is essential for developing therapeutic strategies aimed at stabilizing the neurovascular unit and promoting brain recovery after stroke (76–78).

### 3.6 Gut microbiota modulation

Gut microbiota has emerged as critical modulators of host physiology and are increasingly recognized as therapeutic targets in post-stroke recovery. In this context, “gut health” represents a novel research objective, given its influence on systemic inflammation, metabolic regulation, and neuroprotection (79, 80). Ischemic stroke induces profound alterations in gut microbiota composition and function, a phenomenon increasingly recognized as a contributor to secondary injury and impaired recovery. Stroke triggers systemic inflammation, autonomic nervous system imbalance, and reduced gastrointestinal motility, all of which disrupt microbial homeostasis. Probiotics, defined as live microorganisms that confer health benefits when administered in adequate amounts, have demonstrated potential in modulating gut microbial composition and function. These changes are characterized by a decline in beneficial commensals such as *Lactobacillus* and *Bifidobacterium*, alongside an expansion of opportunistic and pathogenic taxa. The resulting dysbiosis compromises gut barrier integrity, promotes increased permeability (“leaky gut”), and facilitates the translocation of microbial products such as lipopolysaccharide into circulation. *Lactobacillus* and *Bifidobacterium* enhance microbial balance by promoting the expansion of beneficial taxa while suppressing pathogenic species (81, 82). Mechanistically, probiotics reduce ROS production and limit oxidative injury to the gut barrier. They also regulate tight junction proteins, thereby reinforcing epithelial integrity and reducing gut permeability (83, 84). This prevents the translocation of microbial products into systemic circulation, a process that otherwise promotes inflammation and worsens outcomes after stroke. Moreover, stroke-associated microbiota dysfunction disrupts the production of neuroprotective metabolites such as short-chain fatty acids, further weakening host defenses (85, 86). Together, these findings suggest that microbiota dysregulation is not merely a consequence of stroke but an active driver of its progression and long-term sequelae.

Preclinical studies have shown that probiotic supplementation attenuates systemic inflammation, protects against neuroinflammation, and improves both cognitive and motor recovery following ischemic stroke (87, 88). Beyond direct modulation of microbial composition, the gut microbiota regulates lipid and cholesterol metabolism, glucose homeostasis, appetite control, and innate immune responses, all of which intersect with stroke pathophysiology (89).

Prebiotics-non-digestible dietary substrates such as inulin, fructooligosaccharides, and resistant starch-offer an additional avenue for microbiota-based interventions. By selectively stimulating the growth of beneficial bacterial populations, prebiotics enhance the production of short-chain fatty acids (SCFAs), particularly butyrate (90, 91). SCFAs maintain epithelial barrier integrity, reduce microbial dysbiosis, and exert systemic anti-inflammatory effects. Importantly, SCFAs also act on the central nervous system, where they modulate immune signaling and suppress neuroinflammation (92, 93). These properties highlight prebiotics as promising adjunctive strategies for mitigating neuroinflammation and improving functional outcomes after stroke.

### 3.7 Ferroptosis: an iron-dependent form of cell death

Ferroptosis represents a distinct form of regulated cell death driven by iron-dependent lipid peroxidation, playing a critical role in post-ischemic brain injury (94–96). Unlike apoptosis, ferroptosis is characterized by excessive accumulation of lipid-based ROS, particularly in polyunsaturated fatty acid (PUFA)-enriched membrane phospholipids (97). During cerebral ischemia, disrupted iron homeostasis triggers labile iron accumulation through transferrin receptor upregulation and ferritin autophagy (ferritinophagy), catalyzing Fenton reactions that generate hydroxyl radicals and propagate lipid peroxidation (94, 98).

Simultaneously, cellular defense against ferroptosis depends primarily on GPX4 and the cystine/glutamate antiporter system Xc⁻ (99). Ischemia-induced glutamate excitotoxicity depletes cystine availability, compromising glutathione synthesis and inactivating GPX4, thereby removing the critical brake on lipid peroxidation. Neurons are particularly vulnerable due to high PUFA content (DHA, arachidonic acid) and mitochondrial dysfunction that disrupts coenzyme Q10 synthesis (100–102).

Ferroptosis contributes significantly to delayed neuronal death in the penumbral region, extending injury beyond the ischemic core. Experimental inhibition using iron chelators (deferoxamine), lipid peroxidation inhibitors (ferrostatin-1), or GPX4 activators reduces infarct volume and improves outcomes (103–105). Notably, dietary bioactive compounds demonstrate modulate ferroptotic properties through iron chelation, Nrf2-mediated antioxidant activation, GPX4 enhancement, and mitochondrial stabilization (106). This positions ferroptosis inhibition as a promising therapeutic target for dietary interventions during subacute stroke recovery (107, 108).

## 4 Natural bioactive compounds in post ischemic stroke recovery

The multifaceted nature of stroke pathophysiology necessitates therapeutic approaches that can target multiple pathways simultaneously. Given the side effects of western medicine and the invasiveness of external physical interventions, new treatments should be developed. In the past few years, emerging data from both experimental and clinical studies investigated the role of various natural products from medicinal plants and their formulations in the treatment of stroke. Numerous natural products have been identified for the molecular regulation of stroke. In this regard, natural bioactive components offer a unique advantage due to their pleiotropic effects (109, 110). Many of these compounds can modulate stroke pathogenesis and recovery by multiple cellular processes and signaling pathways. This multi-target approach may be particularly beneficial in addressing the complex and interconnected mechanisms underlying stroke-induced brain damage and recovery.

Currently, there are limited commercial drugs specifically approved for promoting neurological recovery after stroke, but several medications are used in clinical practice to support various aspects of post-stroke rehabilitation (Table 1). Cerebrolysin, a neuropeptide-based agent widely used in Europe and Asia, has shown promise in enhancing neuroplasticity and cognitive recovery, though it is not FDA-approved (111, 112). Citicoline (CDP-Choline), another neuroprotective compound, supports membrane repair and neurotransmission and is available as an over-the-counter supplement in some countries (113, 114). For post-stroke depression and motor recovery, fluoxetine, a selective serotonin reuptake inhibitor, has been investigated in clinical trials, though with mixed outcomes (115–117). Additionally, spasticity, a common motor complication, is treated using botulinum toxin A, baclofen, or tizanidine to improve mobility and reduce muscle stiffness (118). Agents such as levodopa and omega-3 fatty acids are also being explored for their potential to enhance motor function and neuro-repair through dopaminergic and anti-inflammatory mechanisms (119, 120). While no drug currently offers a cure for post-stroke disability, these therapies collectively support physical, cognitive, and emotional recovery, underscoring the need for integrated, multi-modal treatment strategies in stroke rehabilitation.

**Table 1:** Bioactive compounds used for post-stroke recovery.

Compound	Primary source	Key mechanisms	*In vitro* evidence	Animal model evidence	Human clinical evidence	Overall level of evidence^*^
Resveratrol	Grapes, berries	Antioxidant, SIRT1 activation, Nrf2 signaling, anti-inflammatory	Robust	Multiple ischemia models (↓ infarct size, ↑ neurogenesis) (126)	Limited / small studies; no large RCT	Moderate (Preclinical-dominant)
Quercetin	Fruits, vegetables	ROS scavenging, anti-inflammatory, BBB protection	Robust	MCAO models show neuroprotection (190)	Very limited clinical stroke data	Moderate (Preclinical-dominant)
Curcumin	Turmeric (*Curcuma longa*)	Anti-inflammatory, Nrf2 activation, anti-ferroptosis effects	Robust	Reduced infarct volume in rodent stroke models (191)	Limited clinical data; bioavailability concerns	Moderate (Preclinical-dominant)
EGCG	Green tea	Antioxidant, mitochondrial protection, Nrf2 activation	Strong	Neuroprotection in ischemic models (146)	Limited stroke-specific trials	Moderate (Preclinical-dominant)
Omega-3 fatty acids (DHA/EPA)	Fish oil, diet	Membrane stabilization, anti-inflammatory lipid mediators, neuroplasticity	Strong	Improved outcomes in stroke models (192)	Some human data (mixed; more prevention than recovery)	Moderate-High
Berberine	Berberis spp., Coptis chinensis	AMPK activation, anti-inflammatory, lipid regulation	Moderate	Reduced ischemic injury in animal stroke model (193)	Minimal stroke-specific clinical evidence	Low-Moderate
Baicalin	Scutellaria baicalensis	Anti-inflammatory, anti-apoptotic	Moderate	Neuroprotection in rodent models (194)	Very limited clinical data	Low-Moderate
Tanshinones	*Salvia miltiorrhiza*	Anti-oxidative, vascular protection	Moderate	Improved cerebral blood flow in rat model (195)	Limited regional clinical use; limited high-quality RCTs	Low-Moderate
Metrnl / FGF21-related metabolic modulators	Endogenous/exercise-related pathways	Metabolic regulation, anti-inflammatory	Emerging	Emerging	Limited stroke-specific data	Low (Exploratory)

* Overall level reflects the balance of mechanistic strength and availability of controlled human data.

Historically, humans have relied on natural foods to protect against disease, largely due to their potent antioxidative, anti-inflammatory, and immunomodulatory properties. These health benefits are attributed not only to essential nutrients such as vitamins and minerals, but also to a wide array of bioactive compounds-molecules that, while not essential for survival, play crucial roles in promoting health and preventing disease. Such compounds are derived from both food sources (like fruits, vegetables, and whole grains) and non-food plants, and include thousands of phytochemicals such as polyphenols, flavonoids, alkaloids, and terpenes. In fact, more than 5,000 distinct phytochemical compounds have been identified in grains, vegetables, and fruits, many of which act as secondary metabolites with therapeutic potential (121, 122). These bioactive substances contribute to the formulation of both traditional and modern medicines, offering benefits in managing conditions such as obesity, diabetes, and cardiovascular diseases through mechanisms involving metabolic regulation, immune support, and antioxidant defense. For instance, fruits are rich in fiber, phenols, and micronutrients like magnesium, calcium, and potassium, all of which support cardiovascular and metabolic health. Importantly, increasing evidence suggests that food-derived and plant-derived natural compounds also exhibit significant biological activity in the context of stroke, aiding in the reduction of neuroinflammation, oxidative stress, and cellular damage. These natural agents may positively influence neural recovery, vascular protection, and cognitive rehabilitation. In this context, we focus on two major sources-food-derived and plant-derived natural bioactive compounds-as promising resources for enhancing post-stroke recovery and improving long-term neurological outcomes.

### 4.1 Food derived small molecules

The potential of food-derived bioactive compounds in post-stroke recovery is further supported by preclinical studies demonstrating their ability to modulate key processes involved in neuroplasticity and functional improvement. For example, Belayev et al. showed that post-stroke treatment with docosanoids, a class of bioactive lipid mediators derived from omega-3 fatty acids, promoted functional recovery, neurogenesis, and angiogenesis in an experimental model of ischemic stroke (123). These findings highlight the potential of dietary interventions or nutraceutical approaches in augmenting endogenous repair mechanisms.

In addition to endogenous metabolites, there is growing evidence supporting the neuroprotective and neuroregenerative potential of food-derived bioactive compounds. These naturally occurring substances, found in various fruits, vegetables, herbs, and other plant-based foods, have been shown to possess antioxidants, anti-inflammatory, and neurotrophic properties that could be beneficial in the context of post-stroke recovery. For example, polyphenols, flavonoids, and other phytochemicals have demonstrated the ability to modulate multiple pathways involved in neuroprotection and neuroplasticity (124, 125). These natural compounds often possess multiple mechanisms of action, including direct antioxidant effects, modulation of endogenous antioxidant systems, and anti-inflammatory properties.

#### 4.1.1 Resveratrol

Resveratrol is a stilbene polyphenol mostly prominent in red grapes, blueberries, and peanuts and in products made from these sources. Common usages for this multifunctional metal chelator are as antioxidant and anti-inflammatory treatments for high cholesterol, cancer, heart disease, and various other diseases. This natural chemical upregulates SIRT1 and Nrf2, promoting neuronal longevity and neuroprotective by its anti-inflammatory, anti-oxidative and anti-apoptotic effects in ischemic stroke (126, 127).

#### 4.1.2 Curcumin

Curcumin is natural polyphenol found in turmeric. It has been studied in a wide range of clinical conditions including cancers, mental health, brain disease, and organ-associated disorders (128, 129). Curcumin has demonstrated anti-inflammatory (130), antioxidant (131), wound-healing (132), and antimicrobial properties (133). Curcumin exerts several beneficial effects toward pre−/post-stroke treatment via its neuroprotective, antioxidant, anti-inflammatory, and anti-apoptotic effects and helps to maintain the integrity of the blood–brain barrier (134). Considering its low absorption and rapid metabolism by oral, some modification is required to optimize its bioavailability, the spherical nano-curcumin micelles have been tested in the clinical trial, the primary result is promising, and further research is warranted (135).

#### 4.1.3 Quercetin

Quercetin, a natural flavonoid widely distributed in plants, has been shown to reduce oxidative stress and promote neurogenesis in animal models of ischemic stroke via antioxidant (136), anti-inflammatory (137), and neuroprotective effects (138). This compound can be found in many plants and foods, including red wine, onions, green tea, apples, and berries. It enhances endogenous antioxidant enzymes like SOD or catalase by the Nrf2/HO-1 pathway, contributing to the mitigation of neuronal damage (139, 140). Quercetin can promote the clearance of senescent cells, which likely contribute to multiple pathways in age-related disorders across multiple systems (141). It is reported that quercetin treatment increased the expression of endogenous tissue kallikrein, which was associated with reduced infarct volume and improved motor function recovery (142).

#### 4.1.4 Epigallocatechin gallate (EGCG)

EGCG is a powerful catechin found mainly in green tea and can be traced to other teas, cocoa products, cranberries, strawberries, prune juice, and other fruits/nuts. Research has primarily involved hypertension, diabetic nephropathy, and certain cancers (143). Some reports have concluded that EGCG can promote weight loss, especially when green tea is combined with dietary adjustments and regular exercise. There are serious side effects if EGCG is taken in high amounts, including liver/kidney failure, dizziness, low blood sugar, and anemia (144). Mechanistically, EGCG scavenges ROS, attenuates lipid peroxidation, and upregulates endogenous antioxidant defenses such as superoxide dismutase and glutathione peroxidase, thereby reducing oxidative stress-induced neuronal damage (145). Additionally, EGCG modulates key signaling pathways implicated in ischemic pathology, including the PI3K/AKT/mTOR axis, NF-κB, and MAPK pathways, resulting in reduced pro-inflammatory cytokine production and suppression of microglial activation (146).

#### 4.1.5 Omega-3 fatty acids

Long-chain polyunsaturated fatty acids comprise the main structure of membrane phospholipid bilayers. Studies have shown how omega-3 fatty acids have protective influences on mitochondrial function and homeostatic regulation of excitotoxicity (147, 148). Variations in the concentrations of omega-3 fatty acids can affect several characteristics, such as membrane fluidity and thickness, protein transport and embedding, and surface receptor signaling and binding. As essential components of neuronal membranes, omega-3 fatty acids help preserve membrane integrity, synaptic function, and signal transduction after ischemic injury (149). Mechanistically, DHA and EPA attenuate post-stroke neuroinflammation by suppressing pro-inflammatory cytokine production, inhibiting microglial overactivation, and promoting the biosynthesis of specialized pro-resolving lipid mediators such as resolvins, protectins, and maresins (142). These lipid mediators actively facilitate inflammation resolution, limit secondary neuronal damage, and support tissue repair.

#### 4.1.6 Vitamin E

Vitamin E, a group of lipid-soluble antioxidants that includes tocopherols and tocotrienols, has been widely investigated for its potential role in ischemic stroke recovery (150). Owing to its strong antioxidant capacity, vitamin E effectively scavenges reactive oxygen species and inhibits lipid peroxidation, processes that are markedly elevated following cerebral ischemia and contribute to neuronal membrane damage and cell death. Beyond its antioxidative effects, vitamin E modulates inflammatory signaling by suppressing NF-κB activation and reducing the production of pro-inflammatory cytokines, thereby limiting secondary neuroinflammatory injury. Experimental studies have shown that vitamin E administration reduces infarct volume, preserves blood–brain barrier integrity, and improves neurological outcomes after stroke (151, 152). In particular, tocotrienols have been reported to exert neuroprotective effects independent of classical antioxidant activity, including the regulation of glutamate excitotoxicity, enhancement of cerebral perfusion, and promotion of neuronal survival pathways (153). Vitamin E also plays a critical role in preserving synaptic integrity and facilitating membrane repair by maintaining lipid homeostasis in neuronal and glial cells (154, 155). Collectively, these findings suggest that vitamin E, especially tocotrienol-rich formulations, represents a promising nutritional adjunct for mitigating oxidative damage and supporting functional recovery following ischemic stroke.

#### 4.1.7 B vitamins (B6, B12, folate)

Vitamin B, particularly folate (vitamin B9), vitamin B6 (pyridoxine), and vitamin B12 (cobalamin), play critical roles in stroke recovery by regulating homocysteine metabolism, neuronal function, and vascular health (156). Elevated homocysteine levels are a well-established risk factor for ischemic stroke and are associated with endothelial dysfunction, oxidative stress, and increased neurotoxicity. Adequate intake of B vitamins lowers homocysteine concentrations, thereby improving cerebral vascular function and reducing secondary ischemic injury (157). Beyond vascular effects, B vitamins support neuronal survival and repair by participating in one-carbon metabolism, DNA synthesis, and methylation processes essential for neuroplasticity and remyelination. Vitamin B12 and folate are particularly important for maintaining myelin integrity and axonal conduction, which are critical for functional recovery after stroke (158). Experimental and clinical studies suggest that B vitamin supplementation may reduce brain atrophy, enhance cognitive recovery, and improve motor outcomes during the post-stroke period, especially in populations with pre-existing deficiencies (159).

#### 4.1.8 Vitamin D

Vitamin D has gained increasing attention for its role in modulating recovery after ischemic stroke due to its pleiotropic effects on neuroprotection, inflammation, and vascular function (160). Vitamin D receptors are widely expressed in neurons, glial cells, and endothelial cells, enabling vitamin D to influence multiple pathways involved in post-stroke injury and repair (160, 161). Mechanistically, vitamin D attenuates neuroinflammation by suppressing pro-inflammatory cytokine production, inhibiting microglial overactivation, and promoting an anti-inflammatory immune phenotype. It also reduces oxidative stress and apoptosis while supporting calcium homeostasis, which is critical for neuronal survival following ischemic injury. In addition, vitamin D contributes to vascular integrity by enhancing endothelial function and maintaining blood–brain barrier stability, thereby limiting secondary damage in the ischemic penumbra (162). Clinical studies have associated vitamin D deficiency with increased stroke severity, poorer functional outcomes, and higher mortality, whereas adequate vitamin D status has been linked to improved neurological recovery (163). Emerging evidence further suggests that vitamin D may promote neuroplasticity, neurogenesis, and synaptic remodeling during the recovery phase.

#### 4.1.9 Caffeine

Caffeine, a widely consumed psychoactive compound, has garnered interest for its potential influence on recovery following ischemic stroke through its neuromodulatory and cerebrovascular effects (164). As a non-selective antagonist of adenosine receptors (primarily A1 and A2A), caffeine modulates neuronal excitability, synaptic transmission, and neuroinflammatory signaling, all of which are perturbed after ischemic injury (165). By inhibiting adenosine A2A receptor-mediated microglial activation, caffeine may attenuate post-stroke neuroinflammation and reduce secondary neuronal damage (164, 166). In addition, caffeine has been shown to influence cerebral perfusion and improve alertness and motor function, which may facilitate rehabilitation and functional recovery during the subacute and chronic phases of stroke (167). Clinical observations indicate that habitual caffeine consumption is associated with reduced stroke risk, but its role in post-stroke recovery remains context-dependent and influenced by dose, timing, and individual susceptibility (168). Collectively, these findings suggest that carefully regulated caffeine intake may support certain aspects of post-stroke recovery, particularly by modulating neuroinflammation and enhancing neural responsiveness, although further studies are needed to define optimal therapeutic windows and dosing strategies.

### 4.2 Plants derived bioactive compounds

**Figure 1:**
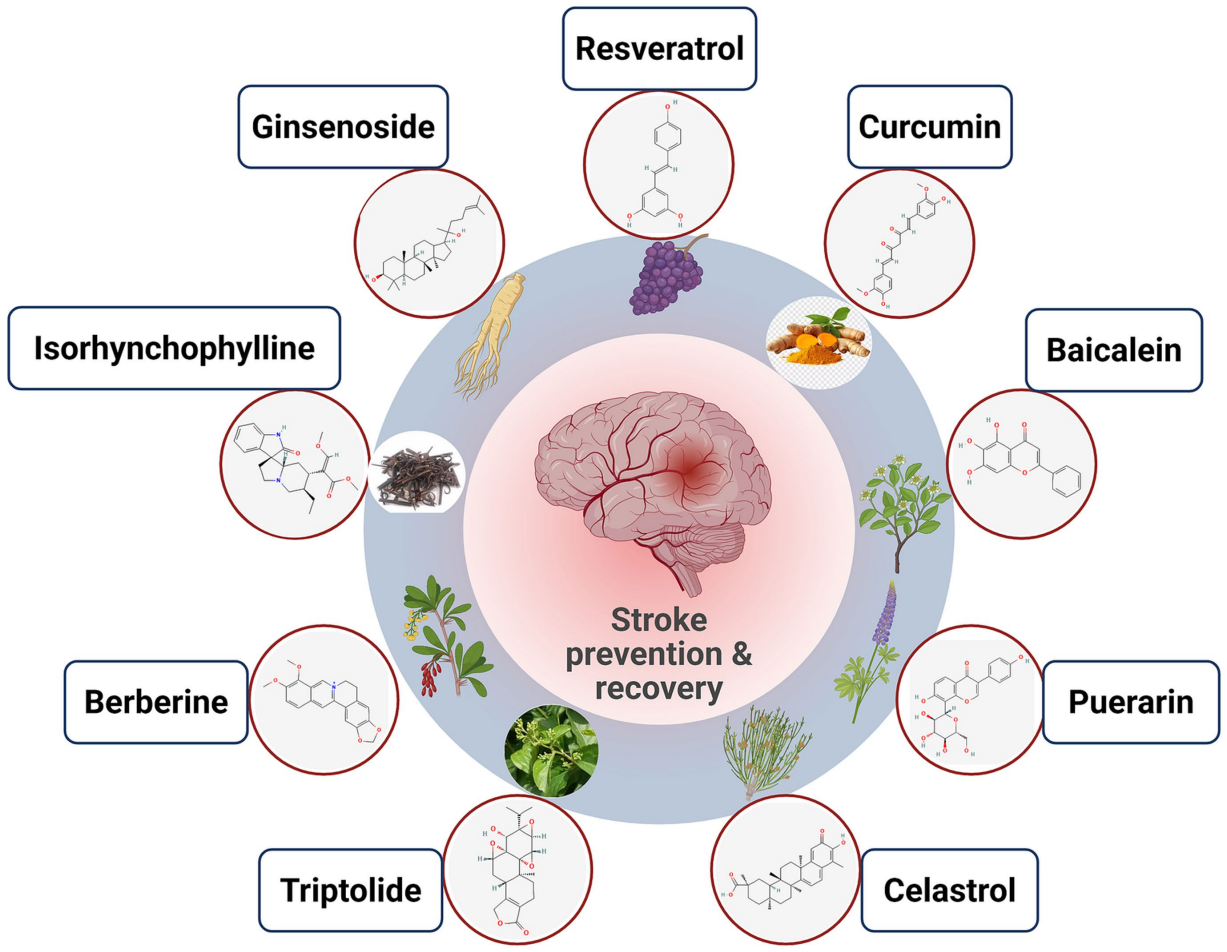
TCM derived compounds for stroke prevention and recovery. Schematic overview of representative plant-derived bioactive molecules with reported neuroprotective and vasculoprotective effects in ischemic stroke. The central panel illustrates cerebral injury associated with stroke and the therapeutic goal of promoting prevention and functional recovery.

Plants have been a rich source of bioactive compounds with therapeutic potential for various diseases, including stroke. Plant-derived bioactive components, also known as phytochemicals, are non-nutritive compounds found in fruits, vegetables, grains, and other plant sources, it has been shown to maintain and improve various health-promoting properties. These compounds include polyphenols, flavonoids, carotenoids, alkaloids, and terpenoids (Figure 1). For example, phenolics and carotenoids, the well-studied bioactive compounds found in colorful fruits and vegetables, showed promising effects in supporting post-stroke recovery (169, 170). Polyphenols such as flavonoids and phenolic acids have shown promise in reducing oxidative stress and inflammation in experimental models of stroke (171). These compounds can act as direct antioxidants by scavenging free radicals and chelating metal ions, as well as indirectly by upregulating endogenous antioxidant enzymes and modulating cellular signaling pathways involved in the oxidative stress response. Similarly, resveratrol, a polyphenol abundant in grapes and berries, has demonstrated neuroprotective effects by modulating multiple pathways involved in post-stroke recovery. A study found that resveratrol treatment enhanced the function of regulatory T cells, which play a crucial role in suppressing excessive inflammation and promoting a pro-regenerative tissue environment in the chronic phase after stroke (172). One example of a plant-derived compound with potential in post-stroke recovery is cordycepin, extracted from Cordyceps militaris. A recent study demonstrated that cordycepin administration ameliorated neurological and cognitive impairments in a mouse model of intracerebral hemorrhage by reducing oxidative stress (173). While silymarin represents a mechanistically plausible adjunct candidate for subacute post-stroke recovery, current clinical evidence remains limited, and further validation is necessary. Its multi-target actions-particularly Nrf2 activation, NF-κB suppression, mitochondrial stabilization, and potential ferroptosis modulation-position it as a promising metabolic-neuroprotective compound warranting rigorous clinical evaluation (174, 175). Table 2 shows the bioactive compounds derived from plants with the potential beneficial effect on stroke.

**Table 2:** Natural bioactive compounds with therapeutic potential for stroke treatment.

Food source	Major bioactive components	Health benefits	Therapeutic applications in stroke
Buckwheat	Rutin, quercetin, D-chiro-inositol, fagopyritols	Potent antioxidant and anti-inflammatory properties; improves endothelial integrity and glucose metabolism (196)	Chronic oral administration of buckwheat polyphenol improved spatial memory deficits in rat stroke models (197)
Maize seed (Corn)	Anthocyanins, phenolic acids (ferulic acid, p-coumaric acid), carotenoids (lutein, zeaxanthin)	Antioxidant, anti-inflammatory, and neuroprotective effects (198)	Higher intake of whole-grain cold breakfast cereals was significantly linked to a lower incidence of ischemic stroke (199)
Olive oil	Hydroxytyrosol, oleuropein, oleic acid, phenolic compounds	Cardioprotective, anti-inflammatory, and lipid-lowering effects; enhances vascular function (200)	Decreases infarct volume, improves cerebral blood flow, and limits neuroinflammation in ischemic stroke models (201)
Eggplant	Nasunin (anthocyanin), chlorogenic acid, flavonoids, phenolic acids	Free radical scavenger; reduces lipid peroxidation; protects neuronal membranes (202)	Prevents neuronal apoptosis and oxidative membrane damage during ischemic events (202)
Soy	Isoflavones (genistein, daidzein), lecithin, saponins	Modulates estrogen receptors, antioxidant and anti-inflammatory effects; improves lipid and glucose metabolism (203)	Enhance neurovascular protection and support neuronal survival post-stroke rats (204)
Blueberries	Anthocyanins, resveratrol, flavonols, chlorogenic acid	Neuroprotective, anti-apoptotic, and antioxidant; promotes cognitive health (205)	Enhance neuroprotective and regenerative effects in rat models (206)
Quinoa	Polyphenols, flavonoids (quercetin, kaempferol), tocopherols, saponins	Antioxidant, anti-inflammatory, and metabolic stabilizing effects (207)	Protects stress-induced hippocampal CA3 dendritic atrophy and improved spatial memory in chronically stressed rats (208)
Milk thistle (*Silybum marianum*)	Silymarin (silybin, silydianin, silychristin)	Potent antioxidant, anti-inflammatory, antifungal, immunomodulatory properties (209)	Reduces oxidative stress, inhibits ferroptosis-associated lipid peroxidation, attenuates neuroinflammation, and improves neurological outcomes in experimental ischemic stroke models (210)

One of the primary mechanisms by which plant-derived bioactive components may contribute to post-stroke recovery is through their potent antioxidant properties. Polyphenols, such as those found in green tea, berries, and various herbs, have demonstrated significant free radical scavenging abilities and can help mitigate oxidative damage in neuronal tissues. For instance, epigallocatechin gallate (EGCG), a major polyphenol in green tea, has shown neuroprotective effects in experimental models of stroke by reducing oxidative stress and preserving mitochondrial function (176, 177). The potential of plant-derived bioactive compounds extends beyond their direct antioxidant effects. Many of these compounds have been shown to modulate multiple pathways involved in the pathophysiology of stroke and recovery. For instance, some phytochemicals can activate the Nrf2 pathway, a master regulator of cellular antioxidant defenses, leading to enhanced protection against oxidative stress. Others have demonstrated the ability to suppress pro-inflammatory cytokines and modulate immune responses, which could help mitigate the chronic inflammation often associated with poor stroke outcomes.

The potential of plant-derived bioactive components to promote neuroplasticity and neurogenesis is another area of active research in the context of post-stroke recovery. Some phytochemicals have been shown to enhance the expression of neurotrophic factors, such as brain-derived neurotrophic factor (BDNF), which plays a crucial role in neuronal survival, differentiation, and synaptic plasticity.

Translating these findings to clinical applications requires further research, including well-designed human trials to evaluate the safety, efficacy, and optimal dosing of these compounds. Additionally, the complex nature of plant extracts, which often contain multiple bioactive components, presents challenges in terms of standardization and quality control. The growing body of evidence supporting the potential benefits of plant-derived bioactive components in post-stroke recovery has also sparked interest in developing novel drug delivery systems to enhance their bioavailability and efficacy.

Natural compounds derived from TCM have emerged as promising therapeutic agents for stroke prevention and recovery. Rooted in centuries of empirical practice, TCM formulations often contain bioactive compounds with potent antioxidant, anti-inflammatory, neuroprotective, and vasodilatory properties. Modern pharmacological studies have identified numerous natural molecules-such as ginsenosides from *Panax ginseng*, baicalin from Scutellaria baicalensis, tanshinones from *Salvia miltiorrhiza*, and curcumin from *Curcuma longa*-that modulate key molecular pathways implicated in ischemic injury, including oxidative stress, apoptosis, mitochondrial dysfunction, and neuroinflammation. These compounds also enhance cerebral blood flow, protect the blood–brain barrier, and promote neuronal regeneration and synaptic plasticity during post-stroke recovery. By targeting multiple cellular and molecular mechanisms simultaneously, TCM-derived natural products offer a systems-level approach consistent with the multifactorial nature of stroke pathology. Integrating these compounds into modern drug discovery pipelines may yield novel adjunct or combinational therapies that complement current pharmacological interventions and improve long-term neurological outcomes.

Berberine can be found in various medicinal plants, common types are *Berberis vulgaris*, *Coptis chinensis*, and *Hydrastis canadensis*. This isoquinoline alkaloid usually accumulates in mitochondria and partially inhibits complex I, which raises the AMP: ATP ratio and transiently activates AMP-activated protein kinase (AMPK). This mechanistic effect can enhance lipid metabolism and insulin sensitivity by heightening fatty acid oxidation and glucose uptake. Berberine metabolically modulates lipid profiles by reducing intracellular cholesterol synthesis and promoting cholesterol efflux. Huperzine A is a natural sesquiterpene alkaloid found in *Huperzia serrata*, used in traditional Chinese medicine for treatments against swelling, fever, and blood disorders (178). This compound works to selectively inhibit acetylcholinesterase and is antagonistic toward NMDA receptors. Primarily, HupA raises synaptic acetylcholine to alter activation-dependent trafficking and recycling of nicotinic α7 and muscarinic M1 receptors. Typically, α7-nAChRs undergo rapid desensitization and internalization, thereby trafficking conditions can determine receptor availability and signaling in neurons and microglia.

Several bioactive compounds demonstrate anti-ferroptosis properties relevant to stroke therapy. Curcumin upregulates GPX4 expression and enhances the synthesis of glutathione, thereby maintaining the cellular antioxidant defense system (179, 180). Resveratrol activates the Nrf2 signaling pathway, promoting expression of ferritin (iron storage) and ferroportin (iron export), which collectively reduce intracellular labile iron levels (181). Quercetin acts as an iron chelator and stabilizes mitochondrial membrane potential, preventing ferroptosis death (182). Omega-3 fatty acids, particularly DHA, reduce membrane lipid peroxidation susceptibility by altering membrane fatty acid composition (183). These mechanisms suggest that targeting ferroptosis through natural compounds represents a promising therapeutic avenue, particularly when combined with traditional neuroprotective strategies.

In conclusion, the exploration of bioactive components from plants represents a promising avenue for advancing stroke prevention and treatment. The diverse mechanisms of action, favorable safety profiles, and potential for multi-target effects make plant-derived compounds an attractive area of research in stroke management. However, further studies are needed to fully elucidate the efficacy, safety, and optimal use of these compounds in clinical settings. As our understanding of the beneficial attributes of bioactive components in stroke continues to grow, it is likely that bioactive components interventions will play an increasingly important role in the comprehensive management of this devastating condition.

## 5 Promising health beneficial attributes of bioactive components

The potential synergistic effects of combining endogenous metabolites and food-derived bioactive compounds represent an emerging frontier in post-stroke recovery research. Interactions between gut microbiota-derived metabolites and dietary components can influence the brain’s metabolic milieu, enhancing neuroprotection and tissue repair. Preclinical and clinical studies have demonstrated that such compounds can improve lipid metabolism, regulate gut health, and reduce low-density lipoprotein (LDL) oxidation. These effects collectively mitigate oxidative stress, modulate endothelial function, suppress inflammation, and prevent platelet aggregation-key factors in stroke prevention and recovery.

**Figure 2:**
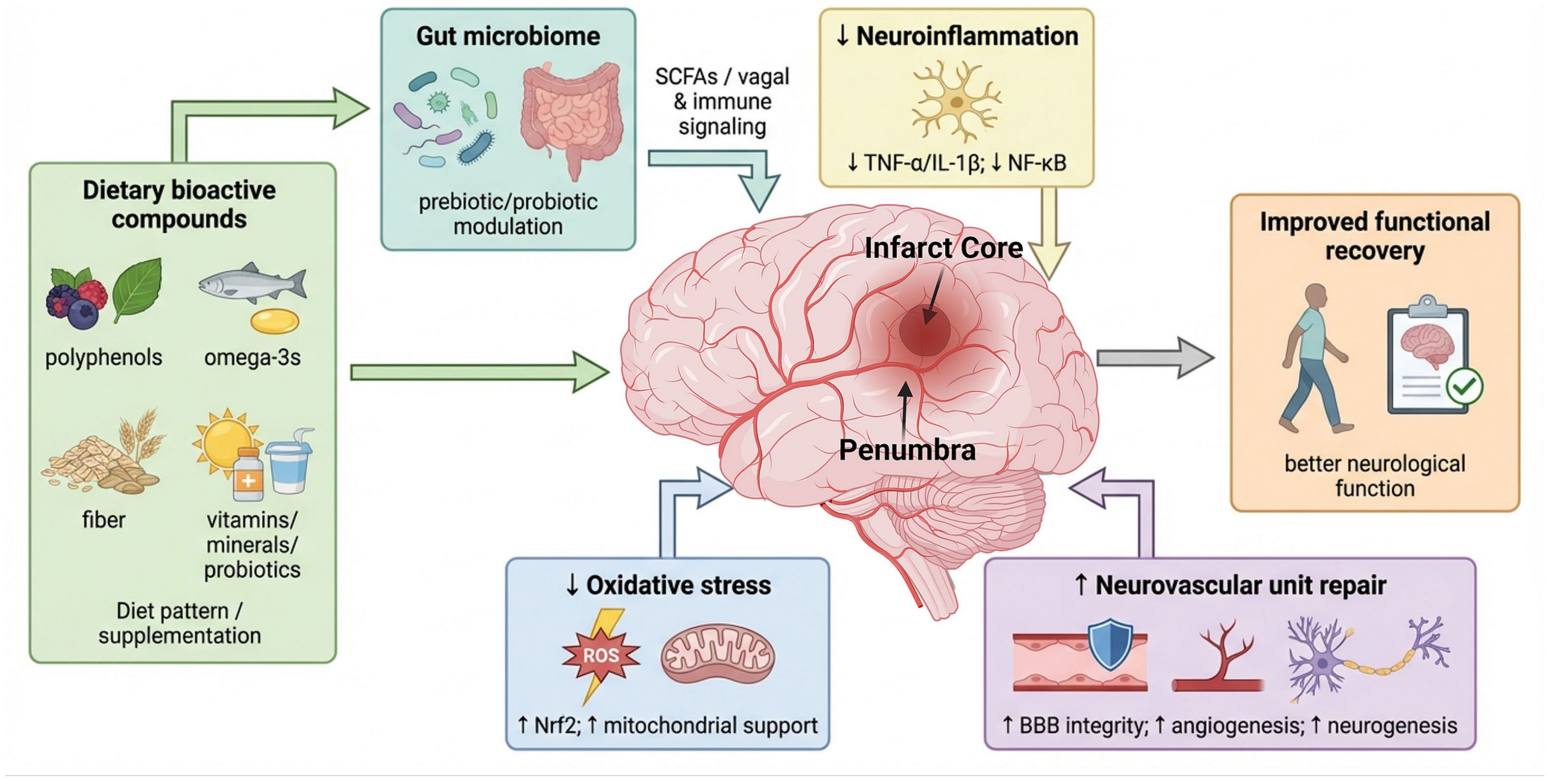
Multidimensional integration of dietary bioactive compounds in post-ischemic stroke recovery. Schematic representation of how dietary bioactive compounds influence ischemic stroke pathology and post-stroke recovery. These compounds modulate the gut microbiome through prebiotic and probiotic effects, enhancing SCFA production and vagal/immune signaling to the brain. In the ischemic brain, characterized by an infarct core and surrounding penumbra, bioactive compounds attenuate key pathological processes, including oxidative stress (↓ ROS; ↑ Nrf2 signaling and mitochondrial support) and neuroinflammation (↓ TNF-α, ↓ IL-1β, ↓ NF-κB activation).

Recent advances in analytical methods, including gas chromatography–mass spectrometry (GC–MS) and high-performance liquid chromatography, have enabled precise characterization of plant-derived bioactive compounds with neuroprotective potential. For instance, GC–MS analysis of *Selaginella dryopteris* identified amentoflavone, a biflavonoid with strong antioxidative and anti-apoptotic effects in models of cerebral ischemia. Similarly, natural molecules such as astaxanthin-a carotenoid found in marine and plant sources-have shown promising roles in promoting neuroplasticity and functional recovery following stroke. Beyond acute neuroprotection, these compounds may support long-term rehabilitation by enhancing synaptic remodeling and cognitive resilience (Figure 2).

Polyphenols and flavonoids from fruits, vegetables, and traditional medicinal plants have been linked to improved cardiovascular health and reduced stroke risk through modulation of hypertension, dyslipidemia, and chronic inflammation. However, translating preclinical efficacy into clinical success remains challenging. Bioavailability, metabolic stability, dosing, and potential interactions with standard therapies require careful optimization. Moreover, patient heterogeneity in age, comorbidities, and genetic background underscores the need for personalized approaches and biomarker-guided interventions. Clinical and epidemiological evidence increasingly supports the relationship between dietary patterns and post-stroke recovery outcomes. Prospective cohort studies have demonstrated that adherence to healthy dietary patterns is associated with improved functional recovery, reduced disability, and lower recurrence rates. The Mediterranean diet has shown particular promise in post-stroke populations. Mediterranean diet was associated with a significantly lower risk of incident stroke in this large UK cohort, with a 17% risk reduction in the highest versus lowest adherence group (HR 0.83, 95% CI 0.74–0.94), and the protective association was primarily observed in women population (184). These benefits were observed after adjustment for traditional cardiovascular risk factors, suggesting independent effects of dietary components on recovery mechanisms.

Nutritional interventions may modulate these metabolic signatures. The majority of nutrigenomic evidence derives from preclinical models, and human studies often lack sufficient sample size, longitudinal design, or mechanistic endpoints. Moreover, pharmacokinetic constraints-including limited bioavailability and uncertain brain penetration-must be considered when interpreting gene-expression effects observed *in vitro*. For example, omega-3 supplementation influences lipid mediator profiles, including specialized pro-resolving mediators derived from DHA and EPA. Polyphenols can alter mitochondrial metabolic flux and reduce accumulation of oxidative metabolites. Metabolomic-guided personalization offers a promising translational pathway. By identifying metabolic phenotypes associated with poor recovery-such as excessive lipid peroxidation or impaired mitochondrial function-clinicians may theoretically tailor nutritional interventions to target specific metabolic vulnerabilities. However, robust validation in prospective clinical studies is required. A clinical trial evaluating concentrated grape juice consumption (high in resveratrol and other polyphenols) in subacute stroke patients reported improved cognitive function and reduced inflammatory biomarker levels at 12-week follow-up. Similarly, green tea consumption (≥3 cups daily) has been associated with better quality of life scores and reduced depression symptoms in stroke survivors in observational studies (185).

The convergence of genomics, epigenomics, transcriptomics, proteomics, metabolomics, and microbiome profiling presents an opportunity to move beyond empirical supplementation toward precision neuro-nutrition. Post-stroke recovery is shaped by systemic inflammation, gut-brain axis interactions, immune reprogramming, and metabolic adaptation. Multi-omics integration may help identify molecular endotypes of stroke recovery and reveal which patients are most likely to benefit from specific dietary bioactive compounds.

Several randomized controlled trials evaluating antioxidant supplementation in post-stroke populations have yielded negative or inconclusive results despite strong preclinical rationale. For example, a double-blinded, placebo-controlled trial investigating oral resveratrol supplementation in patients with acute ischemic stroke failed to demonstrate significant improvements in neurological function, as measured by NIHSS, modified Rankin Scale (mRS), or Barthel Index scores at follow-up. Although resveratrol exhibits robust antioxidant, anti-inflammatory, and SIRT1-activating properties in experimental models, these mechanistic benefits did not translate into measurable functional recovery in clinical settings. These findings underscore the gap between experimental neuroprotection and clinically meaningful neurological outcomes (Table 3).

**Table 3:** Clinically evaluated natural bioactive compounds in ischemic stroke-relevant contexts.

Compound	Human clinical evidence	Typical human dosage (from clinical studies)	Bioavailability and PK challenges	Safety and clinical notes
Resveratrol	Phase II RCTs in cerebrovascular risk populations (reduced markers of inflammation and improved flow-mediated dilation)	150–500 mg/day (oral)	Very low oral bioavailability; rapid conjugation; limited CNS penetration	Generally safe ≤1 g/day; GI discomfort and drug interaction potential (anticoagulants) (211)
Curcumin	Small human trials in cognitive impairment; post-stroke trials limited but anti-inflammatory biomarkers improved	500–2,000 mg/day (often with piperine or enhanced formulations)	Poor aqueous solubility; extensive first-pass metabolism; enhanced by adjuvants	Well-tolerated; rarely GI upset (212)
Ginsenosides (standardized *Panax ginseng* extract)	Multiple RCTs in cerebrovascular insufficiency and post-stroke sequelae (improved neurological scores in some trials)	200–400 mg/day extract	Variable gut microbiome metabolism; active metabolites differ from parent compounds	Generally well tolerated; may alter glucose/blood pressure (213)
Puerarin (isoflavone from *Pueraria lobata*)	Used clinically in China for acute ischemic stroke & recovery (non-RCT and registry data)	~400–800 mg/day (oral/IV standardized)	Low oral bioavailability; better systemic levels via IV	Generally safe in clinical use; monitor for hypersensitivity (214)
Berberine	RCTs in metabolic syndrome and atherosclerosis (stroke risk factors)	500–1,500 mg/day	Poor oral absorption; P-glycoprotein efflux; microbiome transformation required	GI intolerance common; interacts with CYP enzymes (215)
Omega-3 (DHA/EPA)	Large cardiovascular RCTs; subgroup stroke incidence reduction in some trials	1–4 g/day EPA/DHA	Variable incorporation into neural tissue; influenced by baseline diet	Generally safe; high doses increase bleeding risk (216)
Silymarin (Milk thistle; silybin/silibinin-rich extract)	Human trials mainly in liver/metabolic inflammation and oxidative stress biomarkers; direct post-stroke RCT evidence limited/absent	~140–420 mg/day silymarin (often standardized to ~70–80% silymarin; divided doses in many clinical studies)	Low aqueous solubility; variable absorption; extensive phase II metabolism; improved with phospholipid complexes (e.g., phytosome) or enhanced formulations	Generally well tolerated; occasional GI upset/headache; rare hypersensitivity (Asteraceae allergy); potential interactions via transporter/CYP modulation-use caution with anticoagulants/antiplatelets and narrow-therapeutic-index drugs (217)

Similarly, curcumin combined with piperine has shown biochemical efficacy but has limited functional benefit in post-stroke patients. In a randomized clinical trial, supplementation reduced inflammatory markers and improved certain metabolic parameters; however, no significant improvements were observed in functional recovery or quality-of-life measures compared with placebo. This disconnects between biomarker modulation and clinical endpoints highlights several translational challenges, including bioavailability constraints, insufficient central nervous system penetration, heterogeneity in stroke severity, and inadequate trial duration. Collectively, these studies emphasize that antioxidant and nutritional interventions, while mechanistically promising, require more rigorous, biomarker-guided, and adequately powered clinical trials before definitive conclusions regarding their efficacy in post-stroke recovery can be established.

Although natural bioactive compounds show considerable promise in promoting post-ischemic stroke recovery, several translational and clinical challenges must be addressed. As stroke is a pathophysiologically complex and heterogeneous disorder, and the pleiotropic actions of phytochemicals-while advantageous-complicate optimization of dosing regimens, therapeutic windows, and potential drug-nutrient interactions. Clinical evidence further highlights uncertainty regarding the optimal timing of intervention, with some studies suggesting greater benefit during the acute or subacute phase, whereas others emphasize the importance of sustained long-term adherence. In addition, poor solubility, limited BBB penetration, and low oral bioavailability-coupled with significant inter-individual variability in absorption and metabolism-pose substantial barriers to dose standardization and reproducibility. Innovations in drug delivery, formulation chemistry, and systems biology are beginning to address these challenges. Advanced delivery strategies such as nanoencapsulation have been explored to enhance stability, improve pharmacokinetics, and facilitate targeted brain delivery.

Network pharmacology and metabolomic profiling are revealing the complex, multi-target mechanisms by which plant-derived compounds act, providing new opportunities for precision medicine. Despite obstacles, the development of sustainable, plant-based therapeutics offers an attractive and cost-effective complement to conventional stroke care, especially in resource-limited settings. The frequent use of combined dietary patterns or multi-component supplements in clinical studies further obscures attribution of efficacy to specific compounds. Rigorous, well-controlled clinical trials are required to validate safety, define optimal therapeutic parameters, and establish efficacy before widespread clinical implementation.

## 6 Future prospects

**Figure 3:**
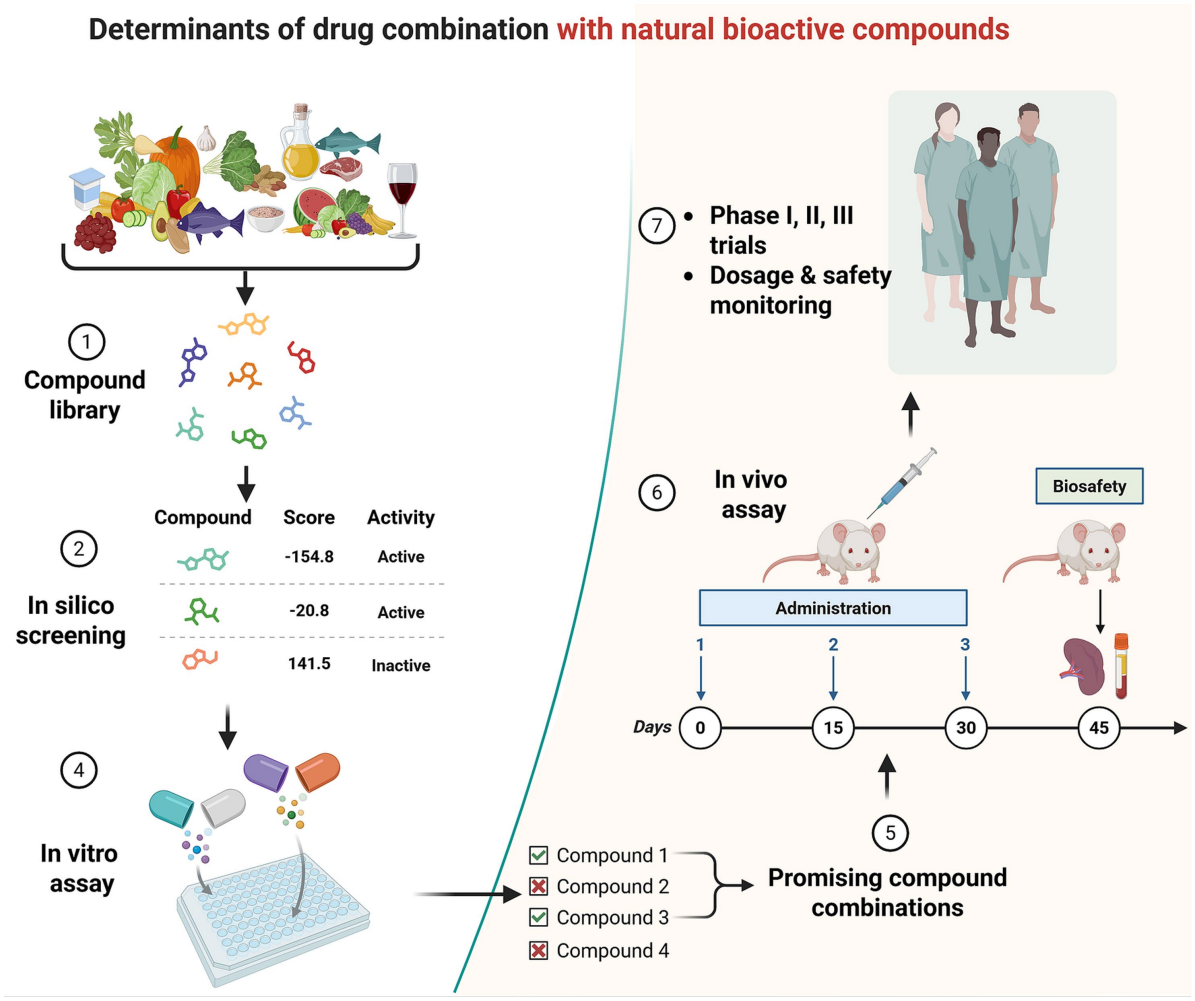
Combination of bioactive components for stroke recovery. This schematic illustrates an integrated workflow for identifying and validating effective drug combinations incorporating natural and food-derived bioactive compounds. The process begins with (1) compound library construction, comprising dietary components, phytochemicals, and natural products. These candidates are subjected to (2) *in silico* screening, where computational modeling and scoring systems prioritize compounds based on predicted activity, target engagement, and safety profiles. Selected hits are then evaluated through (3) prioritization and filtering, followed by (4) *in vitro* assays to assess biological activity, efficacy, and potential synergistic effects. Based on these results, (5) promising compound combinations are identified and advanced to (6) *in vivo* validation, including time-course treatment and functional assessments in animal models. Finally, optimized combinations progress to (7) clinical evaluation, encompassing phase I–III trials with careful dosage optimization and safety monitoring. Collectively, this framework highlights a translational pipeline that bridges computational prediction, experimental validation, and clinical development to guide rational combination therapies leveraging bioactive natural compounds.

Given the multifactorial nature of stroke pathology, combination therapies incorporating natural and food-derived bioactive compounds hold great promise for achieving optimal therapeutic outcomes. Approaches that target multiple biological pathways-such as combining probiotics or prebiotics with dietary interventions, antioxidant supplementation, or pharmacological inhibitors of ROS production-may synergistically restore gut-brain homeostasis. The integration of these natural and food-derived compounds represents a promising strategy for mitigating neuroinflammation, reducing oxidative stress, and promoting post-stroke recovery (Figure 3). Probiotics, prebiotics, dietary modifications, fecal microbiota transplantation, and ROS-modulating agents offer diverse avenues for attenuating excessive ROS production and its detrimental effects. Meanwhile, antioxidants and anti-inflammatory compounds provide complementary mechanisms to counteract oxidative and inflammatory damage within neural tissues.

The emerging field of nutrigenomics provides a mechanistic framework linking dietary bioactive compounds to molecular pathways involved in neuroprotection and neurorepair after ischemic stroke. Rather than acting solely as antioxidants or anti-inflammatory agents, many natural compounds exert their effects through dynamic modulation of gene expression, epigenetic remodeling, and metabolic reprogramming. These multilayered regulatory effects may be particularly relevant in the subacute and chronic phases of stroke recovery, where cellular plasticity, angiogenesis, and synaptic remodeling predominate. From a nutrigenomic perspective, food-derived bioactive compounds exert their neuroprotective effects through several mechanisms: metabolite activities, epigenetic reprogramming, gene-diet interactions, and broad transcriptomic shifts.

The therapeutic effects of dietary compounds are increasingly understood through metabolomic profiling, which reveals how these substances alter cellular metabolite compositions and metabolic pathway activities. Metabolomic analysis has identified specific metabolite signatures associated with improved stroke outcomes, including enhanced mitochondrial metabolism, reduced oxidative stress markers, and normalized neurotransmitter profiles. These findings suggest potential for personalized nutrition strategies based on individual metabolomic responses to specific bioactive compounds. In a prospective study, Yu et al. identified specific serum metabolites associated with ischemic stroke risk, suggesting that distinct metabolic signatures may predict both susceptibility and recovery trajectories (186). Extending such metabolomic analyses to post-stroke populations could facilitate the identification of biomarkers that inform individualized therapeutic strategies-combining metabolic modulation, dietary optimization, and precision supplementation to maximize recovery and long-term neurological outcomes.

Bioactive compounds also influence DNA methylation and histone acetylation patterns, thereby regulating the expression of genes involved in antioxidant defense, inflammation, and neuroplasticity. Ischemic stroke induces widespread epigenetic reprogramming characterized by altered DNA methylation, histone acetylation, and microRNA expressions (187). These changes influence neuronal survival, glial activation, angiogenesis, and neurogenesis. Importantly, several dietary bioactive compounds have been shown to interact directly or indirectly with epigenetic machinery. Polyphenols such as resveratrol and curcumin modulate histone acetylation through regulation of histone deacetylases (HDACs) and sirtuin-1 (SIRT1). Activation of SIRT1 has been associated with enhanced mitochondrial biogenesis, reduced oxidative stress, and improved neuronal survival following ischemia. Similarly, sulforaphane has been reported to influence Nrf2 signaling partly via epigenetic mechanisms, including modulation of histone acetylation and DNA methylation at antioxidant response elements (188). These epigenetic effects may extend beyond acute cytoprotection and contribute to longer-term transcriptional reprogramming that supports repair processes.

Nutrigenomic interactions involving genes such as APOE, MTHFR, SIRT1, and Nrf2 have been implicated in modulating oxidative stress responses, lipid metabolism, and neuroinflammatory pathways. For example, APOE genotype influences lipid transport and neuroinflammation, potentially modifying the response to omega-3 fatty acid supplementation. Similarly, polymorphisms in genes regulating folate metabolism (e.g., MTHFR) may alter homocysteine levels and influence cerebrovascular risk and recovery. These gene-diet interactions highlight the possibility that the efficacy of certain bioactive compounds may depend on underlying genetic architecture.

Beyond single-gene modulation, ischemic stroke induces broad transcriptomic shifts affecting inflammatory networks, synaptic signaling pathways, mitochondrial function, and extracellular matrix remodeling. RNA sequencing studies of fracture hematoma, ischemic penumbra, and peri-infarct tissue consistently demonstrate coordinated changes in Wnt/β-catenin, BMP, mTOR, and NF-κB signaling pathways. Many plant-derived compounds act as natural ligands for nuclear receptors and transcription factors, including Nrf2, NF-κB, and PPARγ. These interactions result in coordinated changes in gene expression programs that enhance cellular stress resistance and promote recovery. Single-cell RNA sequencing studies have revealed that bioactive compounds can induce cell-type-specific transcriptional responses in neurons, astrocytes, and microglia, highlighting the complexity of nutrigenomic effects in the post-stroke brain (189). The expanding body of evidence on food-derived bioactive compounds and endogenous metabolites in post-ischemic stroke recovery presents new opportunities for therapeutic innovation. Understanding the complex interplay between dietary factors, metabolic regulation, and neural repair is essential for advancing stroke management strategies. Future research should focus on translating these findings into clinical practice through the development of targeted nutritional interventions and pharmacological agents that modulate key metabolic and signaling pathways. The use of robust post-stroke recovery medications-ranging from stem cells and neuromodulation to AI-powered rehabilitation and epigenetic interventions-offer real potential cost-effective and life-saving options to promote brain repair and functional restoration. The integration of advanced imaging techniques and metabolomic profiling could further enable real-time monitoring of recovery processes and support the design of personalized therapies based on individual metabolic responses.

Despite these encouraging findings, most supporting evidence to date arises from *in vitro* experiments and animal models. Only a limited number of clinical studies have confirmed the health-promoting potential of these plant-derived bioactive components in humans. Therefore, long-term, large-scale clinical investigations are essential to validate their safety, efficacy, and translational potential before recommending their integration into routine stroke management or neuroprotective dietary interventions. Future research priorities should include: (1) randomized controlled trials with standardized dietary interventions and well-defined stroke subtypes, (2) identification of predictive biomarkers for dietary treatment response, (3) investigation of optimal intervention timing and duration, (4) evaluation of combined dietary and pharmacological approaches, and (5) development of personalized nutrition strategies based on genetic, metabolomic, and microbiome profiles. Integration of nutritional interventions into comprehensive stroke rehabilitation protocols represents a promising avenue for improving patient outcomes.

## 7 Conclusion

In conclusion, the study of natural bioactive compounds and endogenous metabolites in post-ischemic stroke recovery represents a rapidly advancing and translationally relevant area of neurotherapeutic research. Leveraging the synergistic interactions between dietary modulation and intrinsic repair pathways offers a novel strategy to enhance neuroplasticity, attenuate neuroinflammation, restore metabolic homeostasis, and ultimately improve functional outcomes after stroke. Future investigations should prioritize delineating the underlying molecular and cellular mechanisms, refining formulation and delivery platforms to overcome bioavailability constraints, and implementing rigorously designed, adequately powered clinical trials across diverse populations. A deeper mechanistic understanding of the bidirectional interplay among nutrition, systemic metabolism, and neuroregeneration will be essential for developing personalized, mechanism-driven interventions capable of reshaping post-stroke care and rehabilitation paradigms.
